# Supplementary material for: Probing Temperature- and pH-Dependent Binding between Quantum Dots and Bovine Serum Albumin by Fluorescence Correlation Spectroscopy
Source: Nanomaterials (Basel). 2017 Apr 25;7(5):93. doi: 10.3390/nano7050093 (PMC5449974; doi:10.3390/nano7050093)
Supplement: Supplementary file 1 [file nanomaterials-07-00093-s001.pdf]

Probing Temperature- and pH-Dependent Bindingbetween Quantum Dots and Bovine Serum Albumin by Fluorescence Correlation Spectroscopy

Zonghua Wang [1,*], Qiyan Zhao [1,2,3], Menghua Cui [2,4], Shichao Pang [5], Jingfang Wang [6], Ying Liu [3,*] and Liming Xie [2,4,*]

1 Laboratory of Fiber Materials and Modern Textile, the Growing Base for State Key Laboratory, College of Chemistry and Chemical Engineering, Shandong Sino-Japanese Center for Collaborative Research of Carbon Nanomaterials, Collaborative Innovation Center for Marine Biomass Fiber Materials and Textiles, Qingdao University, Qingdao 266071, China; zhaoqy@nanoctr.cn

2 CAS Key Laboratory of Standardization and Measurement for Nanotechnology, CAS Center for Excellence in Nanoscience, National Center for Nanoscience and Technology, Beijing 100190, China; cuimh@nanoctr.cn

3 Laboratory for Biological Effects of Nanomaterials and Nanosafety, National Center for Nanoscience and Technology, Beijing 100190, China

4 University of Chinese Academy of Sciences, Beijing 100049, China

5 School of Life Sciences and Biotechnology, Shanghai Jiao Tong University, Shanghai 200240, China; scpang@sjtu.edu.cn

6 Shanghai Center for Systems Biomedicine, Shanghai Jiao Tong University, Shanghai 200240, China; jfwang8113@sjtu.edu.cn

* Correspondence: wangzonghua@qdu.edu.cn (Z.W.); liuy1@nanoctr.cn (Y.L.); xielm@nanoctr.cn (L.X.);
Tel.: +86-532-85950873 (Z.W.); +86-186-0020-3627 (L.X.)

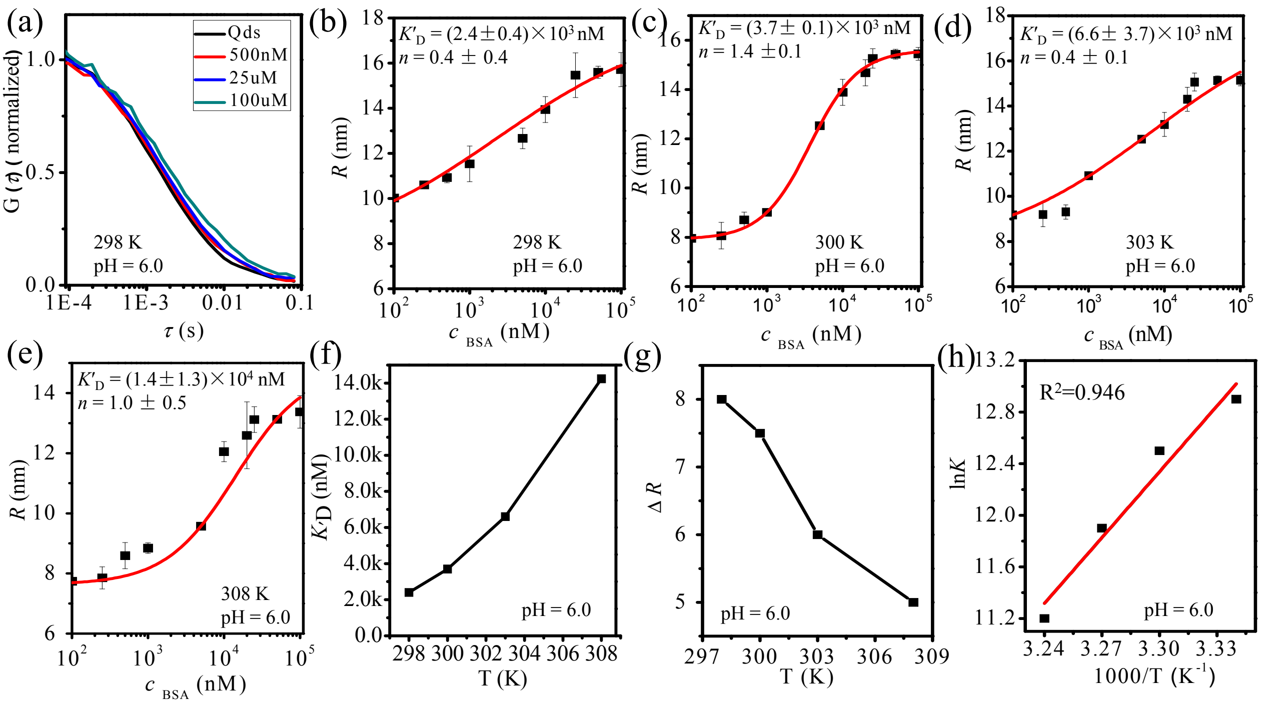

Figure.S1 (a) FCS correlation curves and (b) hydrodynamic radius of QDs-COOH at different BSA concentrations (pH = 6.0) at 289 K. (c-e) Hydrodynamic radius of QDs-COOH at different BSA concentrations (pH = 6.0) at different temperatures. (f-g) Plot of K'_D, ΔR against temperature. (h) Plot of ln$K$ against 1/T.

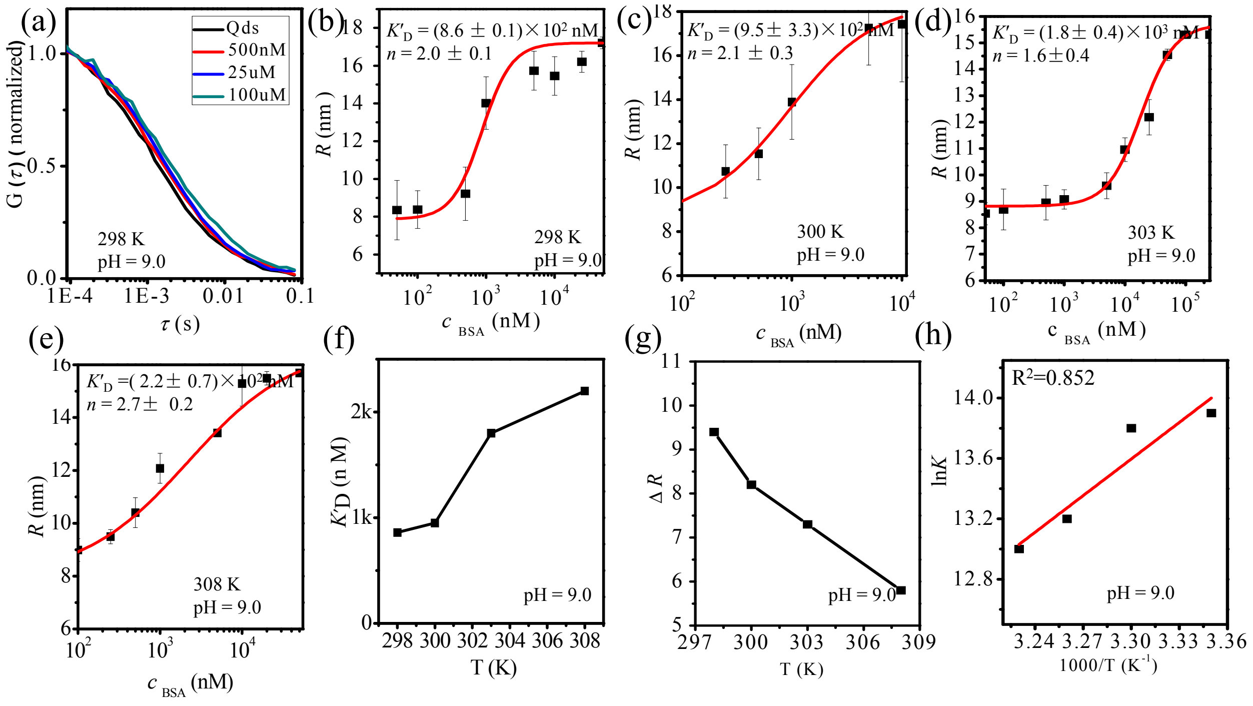

Figure. S2 (a) FCS correlation curves and (b) hydrodynamic radius of QDs-COOH at different BSA concentrations (pH = 9.0) at 289 K. (c-e) Hydrodynamic radius of QDs-COOH at different BSA concentrations (pH = 9.0) at different temperatures. (f-g) Plot of K'_D , ΔR against temperature. (h) Plot of ln$K$ against 1/T.

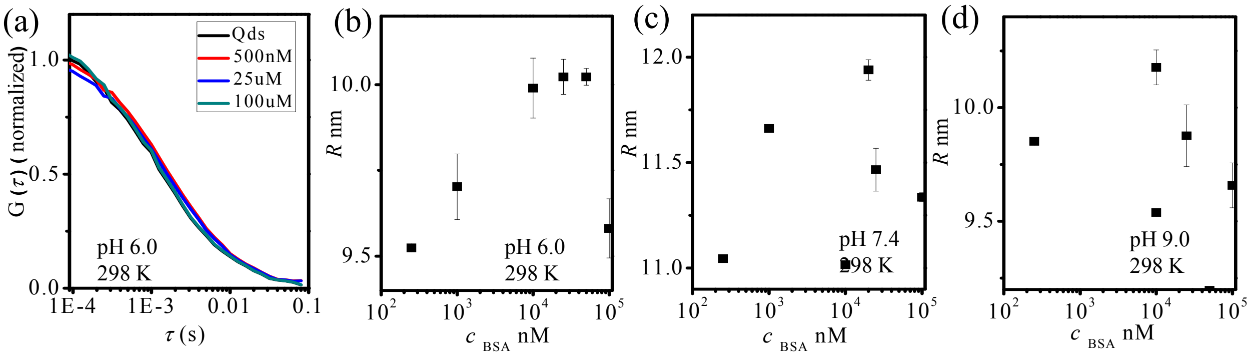

Figure. S3 (a) FCS correlation curves and (b-d) Hydrodynamic radius of QDs-PEG at different BSA concentrations (T = 298 K) at different pH.
